# Medical Gossip

**Published:** 1862-01

**Authors:** 


### Art. X.—MEDICAL GOSSIP.

The opening of the Session was characterized, as usual, by a pleasant variety in the matter and mode of treatment of the Introductory Lectures with which it is customary to preface the real work of the Schools. Dr. Frankland, at St. Bartholomew's, urged the peculiar importance of the study of the sciences collateral with medicine, illustrating his theme more especially from chemistry and physics. It was not sufficient, he observed, to know these sciences merely in their technical applications to medicine; for the dignity of the profession, as estimated by the community, was not a little dependent upon a knowledge of the bearing of these sciences "upon those natural phenomena in the outer world which constantly attract the attention of the observant, and occasionally even of the most ignorant portion of mankind." Mr. Canton, at Charing Cross, overflowed with excellent advice on the special subjects of medical studies. Mr. Masters, at St. George's, referred to the peculiar nature of the profession, its rewards, and the mode of securing them by the conduct of the practitioner towards the profession and the public, and touched upon the "art of managing his patient," remarking,—"The favourable verdict of the public was to be gained not so much by professional attainments, as by matters of which the public are competent judges, such as the general information and character of the practitioner, and the esteem in which they see him held by his fellow-labourers. The goodwill of his brethren was obtained by his special skill and knowledge, and by his conduct towards themselves." The latter sentences remove the somewhat Mephistophelian effect produced by the phraseology of the first sentence. In addressing the Scholar, Mephistopheles told him that it was wasted time to take science as a guide in Medicine; for, he said,—

> "Each one you meet will only trace
> And learn the parts he easiest can.
> Who can the passing moment take,
> And of it all advantage make,
> Him you will find the proper man."

Mr. Masters also reviewed the educational measures of the Medical Council, and discussed the importance of a study of the natural sciences in medicine. Dr. Webb, at Grosvenor-place, discoursed eloquently on habits of working as the only means by which success was to be gained, upon biological science as the only sure foundation of practical medicine, and upon the dignity of medicine. Dr. Odling, at Guy's, ably descanted on the "certainties of medical science," and the need of studying medicine in a philosophical spirit. "The medical man," he said, "had to exert his scientific judgment so as to interpret rightly the phenomena of disease presented to him, to foresee the course it would take, and the conditions to which it would give origin; and, guided by his knowledge of the natural history of the disease, of what it was and what it would be, had to exert his artistic judgment as to how he should act. But there was a still higher function for the judgment of the philosophic physician, namely, the interpretation, not of some particular case of disease, but of disease in general, so as to effect an advancement of medical knowledge." Professor Bentley, at King's, abounded in good advice; and Dr. Ramsbotham, at the London Hospital, proposed to give "a plain, practical address," which, however, proved to be a singularly able and eloquent disquisition on the duties of lecturer and student. Mr. Spencer Smith, at St. Mary's, dwelt mainly upon those motives which might have, and those which *ought* to have, led to a choice of medicine as a profession, painting the physician in his noblest character. Let the student listen, he said,—

"—To Francis Bacon, the spiritual father of modern science, to whose philosophy they owed medical and sanitary science, and who owed his philosophy to his Christianity. 'But,' says he, 'the greatest error of all the rest is the mistaking or misplacing of the last or furthest end of knowledge; for men have entered into a desire of learning and knowledge, sometimes upon a natural curiosity, and an inquisitive appetite; sometimes to entertain their minds with vanity and delight; sometimes for ornament and reputation; sometimes to enable them to victory of art and contradiction, and most times for art and profession, and seldom sincerely to give a true account of their gift of reason to the benefit and use of men; as if there were sought in knowledge a couch whereupon to rest a searching and restless spirit, and a terrace for a wondering and variable mind, to walk up and down with a fair prospect, or a tower of state for a proud mind to raise itself upon, or a fort commanding ground for strife and contention, or a shop for profit and sale, and not a rich storehouse for the glory of the Creator and the relief of man's estate.' Here, then, was the true motive principle declared. Theirs was really to be 'a calling.'"

Dr. Murchison, at the Middlesex, devoted himself to an able and elaborate examination of the student's duty and position, teaching that medicine was to be prosecuted for its own sake, and ended an eloquent peroration by saying:—

"Ever bear in mind that your calling is a sacred one—that it is your blessed privilege to imitate the example of the Great Physician, and to go about the world doing good. Not a day will pass but you will have it in your power to alleviate suffering, to heal the sick, and to minister consolation to the dying. But be careful not to incur the charge of abusing these great privileges. Let not familiarity with disease and death, and the frail emblems of mortality, render you callous to the effects which they naturally produce upon the mind. When you are called upon to witness the flickering and extinction of the flame of life, remember that ere long you yourselves will be the principal actors in such a scene, and that then you will have to account for the privileges and talents entrusted to you on earth. Think what that account must be if you make the sacred privileges of your calling and the sufferings of humanity entirely subservient to the attainment of wealth and worldly distinction. Above all, 'Seek ye first the kingdom of God and his righteousness,' and be assured that 'all these things shall be added to you.'"

Dr. Bernays, at St. Thomas's, ably treated of the requirements and duties of the physician, touched on the chief quackeries of the day, and the just relation of the profession to the Legislature. Professor Harley, at University College, dealt mainly, and most admirably and philosophically, with the aspect of Medicine as a true experimental and inductive science. "We have" said he, at one portion of his lecture—

"—Arrived at that stage of advancement that we have not only the power of imitating, but also, at pleasure, of calling into existence, certain diseases. Thus, for example, by injecting a few drops of alcohol into the portal vein, we can give an animal diabetes; by puncturing a portion of the medulla oblongata, albuminuria; or by pinching the vagus, palpitation of the heart. And not only can we produce these, the symptoms of disease, but even morbid changes themselves. Thus, by directly acting on the pneumogastric nerve, cough, dyspnœa, and increased bronchial secretion can be induced on the one hand, and, on the other, the anatomical lesions of pneumonia and pleurisy. So again, by exciting the solar plexus, we can bring on diarrhœa and dysentery, together with the structural changes which habitually accompany them. Nor is this all. We have it in our power even to induce diseases of a more general nature; for with a drop or two of acid, we can call into existence rheumatism and heart disease; or with a little decaying organic matter, bring on fever and death. Then again, as far as surgical diseases are concerned, there is scarcely one which we cannot produce artificially, no matter whether it be a cataract in the eye or a stone in the bladder. In fact, the further we advance, the more clear does it become that man has it in his power, not only to produce morbid symptoms, but even actual diseases, with their complete chain of results. And lastly, as regards the treatment of disease, that is now no longer a happy knack acquired by accident in the field of chance. The physiologist knows the almost miraculous power he possesses in the materia medica. Astronomy has boasted of its wonders; but they are nothing, in my opinion, compared to the wonders of physiology. Who ever could have believed that the 'story of a dead heart' could be anything but a stage fiction? Yet, strange as it may appear, it is a scientific reality. The physiologist has it in his power, by means of two poisons, to produce the wonderful sight of a dead heart in a living body, as well as that of a dead body with a living heart. Now and then we meet with men, even in the ranks of our profession, who say they do not believe in the power of remedies. Such men most probably began with exaggerated notions of the power of drugs, and, by injudiciously employing them, failed in obtaining the expected results; and, jumping to the opposite extreme, deny their use altogether, forgetting that their want of success may be due to the frailty of their knowledge rather than to the inefficiency of the medicine. Even with common remedies what mighty effects can be produced! We can excite the brain to increased action with belladonna; we can lull it to repose with cannabis indica. We can stimulate a motor nerve with strychnine so as to throw all the muscles of the body into one fearful tetanic spasm; or we can paralyse them with conium, and render them flaccid as in death. We can hasten the rapidity of the bounding pulse with hellebore; we can slacken its speed with aconitine. In fact, gentlemen, we have it in our power to produce at pleasure almost every conceivable action and counteraction on the human body."

Finally, Dr. Marcet, at Westminster, discoursed on the qualifications for the satisfactory study of medicine, the methods adapted to carry on original investigations, and gave the student much excellent advice for his guidance.

We regret very much to observe that the Admiralty have of late given cause for a very general feeling of dissatisfaction among the officers of the Medical Department of the Navy, by a promotion which has very much the semblance of a prostitution of that patronage which, for the benefit, well-being, and contentment of the service, should be fairly and judiciously administered.

From the *Daily News* we learn that Dr. Robert Beith, a Surgeon in the Royal Navy of only nine years' standing, and who has of late and with an unwonted degree of rapidity filled a succession of offices, usually regarded, as far as surgeons' appointments are concerned, among the prizes of the service, has not only been promoted to the rank of Deputy Inspector-General, but has also been appointed as such to Plymouth Hospital. It is not alleged that the promotion was unjustly bestowed; but the peculiar grievance of the case, as represented, and we think with great reason, is the fact of Dr. Beith's being appointed to a home hospital in preference to others who have been serving in hospitals on foreign stations. It is, we believe, the usage in the service, departed from only in the case of old officers, that a medical officer promoted to inspectorial rank relieves an officer of the same grade serving abroad.

This wholesome rule has not been observed in the present instance, to the prejudice and disappointment of those on foreign stations, each of whom had the right to expect that this vacancy at Plymouth, if it did not call him home, would, at all events, advance him a step in that direction.

Dr. Armstrong of Malta Hospital, Dr. Smart of Bermuda, and Dr. Mackay of the Hospital at Hong Kong, are alluded to as being more particularly aggrieved by the appointment in question.

The *Daily News* concludes a very appropriate leading article by observing that "it will be in vain for such statesmen as the late Lord Herbert, General Peel, and Sir John Pakington to devise rules for the just elevation of the medical department of the army or navy, if patronage is to be disposed of, so as to cast abroad the elements of doubt, discontent, and uncertainty amongst its officers."

The terrible famine which committed such great devastation in the North-West Provinces of India, in the past year, was followed by an equally terrible outbreak of cholera, in July and August last. The ravages of the epidemic were greatest in Lahore. In less than seven weeks 500 European soldiers were carried off. From an interesting report on the attack of epidemic cholera in Agra and Central India, in 1860, by Dr. John Murray, Deputy Inspector-General of Hospitals, published in the *Indian Annals of Medical Science*, we learn that during the past eighteen years there have been four outbreaks of cholera in the North-West Provinces. "It appeared with great intensity at Cabool in 1842, under the name of 'Black Death,' and advanced steadily in a southerly direction during the hot and rainy seasons, being invisible during the cold season. It reached Peshawur in 1843, Lahore in 1844, Umballa, Soobathu and Meerut in 1845, Gwalior in 1846, Indore in 1847. From this it crossed the Nerbudda into the Deccan." The second attack was like a recurrent wave, and appeared at Indore in 1849, Gwalior in 1850, Agra, Meerut and Nerbudda in 1851. The third came from the East, and prevailed with great severity at Benares in 1855, along the foot of the Nepaul Hills in the spring of 1856, and at Allahabad in the hot season the same year. In July, after the rains set in, the disease appeared at Agra, and radiated along the commercial routes to the West, through Bhurtpore to Ajmere, and to the North through Muttra, Meerut, Umballa, and Lahore. The attack of 1860 also came from the East, like the preceding one. Cholera prevailed at Allahabad at the commencement of the hot season, extended to Oorai in June, and Gwalior 22nd July—some days after the first fall of rain. It appeared at Agra in the beginning of August, and a few days later at Muttra, beyond which it did not extend. The European troops suffered most.

A Special Commission, consisting of Mr. Strachey of the Civil Service; Dr. Linton, C.B., and Dr. McClelland, Inspectors-General of Hospitals; Lieut.-Col. Gawler, 73rd Regt., and Major P. Stewart, Bengal Engineers, has been appointed to inquire into the recent outbreak among the European troops.

Dr. Murray tells an interesting story of the effects of anticipatory measures in preventing cholera. He caused the old-fashioned cholera pill, composed of *opium, black pepper,* and *assafœtida,* to be distributed among the men in the infected stations, directing that a pill should be taken by every man whose *bowels were open during the night.* By an amusing error at the Fort of Muttra, the sergeant-major *served a pill to each man to be taken with his grog!* This created a laugh at the station; but, curious enough, there was not another case of cholera in the fort for a fortnight. The hint was acted upon, and with the best results—only one fatal case occurring in the camp afterwards.

No more graceful or fitting tribute could be given to the memory of the lamented Lord Herbert, than that which would identify his name for ever with the great sanitary and medical reforms in the army, which he so laboriously, but effectively, brought about. To him, as Mr. Gladstone pointed out at the "Memorial Meeting" held in November last, we are chiefly indebted for the Commission for inquiring into Barracks and Hospitals, for the re-organization of the Medical Department, and for the Commission for inquiring into and remodelling the Medical Education of the Army, also for presenting to the public the vital statistics of the army, and for the General Hospital at Woolwich. In consequence of the measures in which Lord Herbert took so commanding a share (at so great a cost to himself), it may be said that the mortality of the army has been reduced one-half. At the meeting we have referred to (in the course of which Mr. Gladstone took occasion to pay a just meed of praise to the medical staff of the army, in bringing about the reforms which Lord Herbert's influence had happily effected), it was proposed—"That a subscription be raised for the purpose of erecting a statue to the late Lord Herbert, and also for the endowment of exhibitions or gold medals in connexion with the Army Medical School at Chatham, to be given at the end of each course of instruction to the candidate or candidates for admission who evince the highest proficiency in the knowledge of the art of preserving the health of the troops at home and in the field." The proposition was unanimously agreed to, and is now being carried into effect.

The new model Military Hospital on Redbrook Common, near Woolwich, is to be termed, by command of Her Majesty, the Herbert Hospital. Further, it is proposed to found a "Herbert Convalescent Hospital." This proposition originated at Salisbury, which city also purposes to erect a local memorial in form of a statue. Numerous subscriptions have been received in furtherance of both objects.

In April last, a deputation of the London Society for the Prevention of Cruelty to Animals, in a private audience readily granted for the occasion, prayed the intervention of the Emperor Napoleon in putting a stop to the system of vivisection carried on in the Veterinary Colleges of France, and more particularly at Alfort. The deputation also directed the Emperor's attention to the subject of vivisection proper, and asked his aid in an investigation into its existence, and the excuse for its practice under the plea of its necessity, which was challenged by the Society. Shortly after the deputation, vivisection was prohibited in the veterinary colleges until further notice, and the directors of the colleges at Alfort, Lyons, &c., were directed to make reports to the Emperor on the subject. The Emperor also submitted the question of "vivisection" to the Academy of Medicine for consideration, and the Academy has nominated a commission to inquire into the matter, consisting of MM. Cruveilhier, Cloquet, Claude Bernard, Robile, Moquin-Tandon, and Leblanc. The opponents of the practice of vivisection contend, according to the Paris correspondent of the *Times*, "that in every light of humanity 'vivisection' must be viewed as inadmissible unless an absolute necessity for it exists; that necessity they cannot admit, and they challenge proof it. Many of the deductions of Majendie were, they say, the introduction of so many grave evils in a series of faulty and false conclusions, the consequences of fallacious and unnatural premises; that the very principle of vivisection is in itself essentially vicious, being inseparable from the displacement and disturbance of the functions of many organs in the process of arriving at information as to others; the very violence of the operation itself sets up an artificial or unnatural state of things wholly useless for safe or reliable conclusions; and they ask, 'if Majendie and his school have practised it with such successful and useful results, whence comes it that the same experiments and researches have to be re-enacted ?' Surely, the results have been chronicled and noted, and when and at what point are these researches to terminate ? And even could a necessity appear to be established, then the interests of humanity, binding on all, demand that any recourse to the practice should be placed under the severest regulations, and regarded as an 'exception,' and not as a 'rule,' until public opinion shall ultimately denounce it."

The Academy Commission has not yet reported, but there is not much difficulty in surmising what that report will be as respects the scientific bearing of the question. The deputation of the Society for the Prevention of Cruelty to Animals would appear to have hopelessly confounded the alleged barbarities of the French veterinary colleges with the practice of vivisection in scientific researches, and to have taken little or no care to make themselves acquainted with the results and actual facts of the latter. The quotation we have given from Professor Harley's Introductory Lecture at University College is a sufficient reply to the crude observations of the deputation on the scientific merits of the question; and the recent researches of the same gentleman on the pathological effects of slow-poisoning would suffice to clench the value of vivisection, if clenching be needed.

---

